# Stochastic Optical Reconstruction Microscopy Imaging of Microtubule Arrays in Intact *Arabidopsis thaliana* Seedling Roots

**DOI:** 10.1038/srep15694

**Published:** 2015-10-27

**Authors:** Bin Dong, Xiaochen Yang, Shaobin Zhu, Diane C. Bassham, Ning Fang

**Affiliations:** 1 Ames Laboratory, US Department of Energy and Department of Chemistry, Iowa State University, Ames, Iowa 50011; 2 Department of Genetics, Development and Cell Biology, Iowa State University, Ames, Iowa 50011; 3 Plant Sciences Institute, Iowa State University, Ames, Iowa 50011; 4 Department of Chemistry, Georgia State University, P.O. Box 3965, Atlanta, Georgia 30302

## Abstract

Super-resolution fluorescence microscopy has generated tremendous success in revealing detailed subcellular structures in animal cells. However, its application to plant cell biology remains extremely limited due to numerous technical challenges, including the generally high fluorescence background of plant cells and the presence of the cell wall. In the current study, stochastic optical reconstruction microscopy (STORM) imaging of intact *Arabidopsis thaliana* seedling roots with a spatial resolution of 20–40 nm was demonstrated. Using the super-resolution images, the spatial organization of cortical microtubules in different parts of a whole Arabidopsis root tip was analyzed quantitatively, and the results show the dramatic differences in the density and spatial organization of cortical microtubules in cells of different differentiation stages or types. The method developed can be applied to plant cell biological processes, including imaging of additional elements of the cytoskeleton, organelle substructure, and membrane domains.

The emergence of far-field super-resolution microscopy techniques^1^,^2^ has provided researchers with new opportunities for further insights into subcellular structures. The diffraction limit for light microscopy of about half of the wavelength of light is overcome in super-resolution techniques through spatial or temporal modulation of fluorophores. A group of techniques, named stochastic optical reconstruction microscopy (STORM)^3^,^4^,^5^, photoactivated localization microscopy (PALM)^6^, and fluorescence photoactivation localization microscopy (FPALM)^7^, relies on the stochastic nature of single molecule switching. Photoactivatible fluorophores are switched randomly between a fluorescent state (on-state) and a dark state (off-state) or any other form that is non-fluorescent at the same wavelength, and isolated fluorescent molecules are localized by fitting with a point spread function (PSF) or with a Gaussian function as a close estimate. The enhancement of the spatial resolution using these techniques depends on the precision with which individual fluorescent molecules can be localized. This is in reverse relation to the square root of the photon number detected from a single molecule burst^8^,^9^. Therefore, single molecule detection with sufficiently high signal-to-noise ratio (S/N) is commonly required to achieve nanometer-scale localization accuracy. Total internal reflection (TIR) illumination was adapted to meet such requirements. Its thin illumination volume (a few hundred nanometers from the interface) greatly reduces the out-of-focus background. Clearly, however, this also restricts the imaging depth. Various strategies, such as combining epi-excitation and two-photon activation or using multiple imaging planes simultaneously, have been demonstrated to extend the super-resolution imaging depth to whole cell and tissue samples^10^,^11^,^12^,^13^.

Nearly all of these advances in super-resolution imaging were performed with mammalian cells. Very few reports exist of the study of cellular structures with such high resolution in plant samples due to numerous technical challenges^14^, including the generally high fluorescence background due to significant autofluorescence of endogenous components, and the presence of the cell wall (>250 nm thickness). The former leads to low S/N for single molecule detection and therefore low localization accuracy and low spatial resolution. The latter contributes to a higher background due to additional layers with mismatched refractive indices (causing more severe scattering and spherical aberration) and restricts the use of TIR illumination.

Several super-resolution imaging techniques have been tested for imaging plant samples. The structure of perinuclear actin in live tobacco cells was visualized with a lateral resolution of 50 nm by combining PALM imaging with optical sectioning^15^. The organization of cellulose microfibrils on the outer side of the cell wall in live onion epidermal cells has been studied by STORM imaging with a lateral resolution of 100 nm^16^. Structured illumination microscopy, which uses specially designed illumination patterns to spatially modulate fluorophores^17^, was used for imaging the dynamics of endoplasmic reticulum, plasmodesmata, and cortical microtubules in live cells with a two-fold improvement in the spatial resolution (~100 nm) over traditional fluorescence microscopy techniques^18^,^19^. Stimulated emission depletion (STED) microscopy has also been used to measure the size of protein clusters on the lateral plasma membrane of plant cells with a lateral resolution of 70 nm^20^. Despite all of these recent advances, imaging cellular structures deep in plant cells, such as those of intact Arabidopsis root tips, with a spatial resolution below 50 nm remains a challenge.

Plant cells have highly anisotropic shapes that are important to cell function and multicellular development^21^. The cortical microtubule array is one of the key factors in determining plant cell morphogenesis. In rapidly expanding plant cells, cortical microtubules are often densely aligned parallel to each other with a transverse orientation to the direction of growth. Several models including the cellulose synthase constraint hypothesis^22^,^23^,^24^, templated-incorporation model^25^, and the microfibril length regulation hypothesis^26^ have been established or proposed to explain the role of cortical microtubule arrays during cell expansion. Detailed quantitative information on the structure and organization of cortical microtubules is critical to an understanding of the mechanism of cell expansion and directional growth. We therefore use the cortical microtubule array as a test case in the present study to develop techniques for super-resolution imaging within whole-mount seedling root tips. We successfully demonstrated a spatial resolution of 20–40 nm in whole plant tissue imaging by combining direct STORM^27^, which is essentially STORM without an activator fluorophore, with variable angle epi-fluorescence microscopy (VAEM)^28^,^29^. Such high spatial resolution is crucial to resolve the dense cortical microtubule structures in the active elongation zone of the plant root. Quantitative data including microtubule density and orientation have been obtained for intact Arabidopsis seedling roots.

## Results and Discussion

### Comparison of the background between epi-illumination and VAE illumination

The evanescent field generated by TIR illumination excites samples within a few hundred nanometers of the solid-liquid interface^30^. The thin illumination volume largely rejects the out-of-focus background, thereby offering an ideal optical sectioning method for imaging with high S/N, and has been frequently used for super-resolution imaging in animal cells^3^,^6^. However, the application of TIR illumination is restricted to the surface because of its shallow illumination depth, limiting its use with plants due to the presence of the thick cell wall. To overcome this limitation, VAE illumination^31^ was developed to extend the illumination depth to a few tens of micrometers when the incident angle is operated at subcritical angles (smaller than but still very close to the critical angle), while the S/N is still several fold higher than that of epi-illumination (Supplemental Fig. 2B,C). The features of VAE make it an excellent choice for imaging cortical microtubules in plant root cells.

Cortical microtubules of young Arabidopsis seedling root cells were labeled with the photoswitchable dye Alexa Fluor 647 through immunostaining with tubulin antibodies. The excitation (660 nm) laser and the activation (405 nm) laser were collimated and operated at subcritical angles at the interface of the root sample and the coverslip (Supplemental Fig. 1 and Supplemental Fig. 2). The background is greatly reduced with the confined excitation volume under VAE illumination (Supplemental Fig. 3). The cross-sectional profiles along the elongation direction of the root cells show that dense microtubules are much better resolved in images under VAEM than under epi-illumination (Supplemental Fig. 3E). The light intensity profile on the sample side is shown in Supplemental Fig. 2D. Within 10° of the critical angle, the light intensity is higher than the incident beam. This feature is also important for STORM imaging experiments, in which turning off fluorescent molecules to a ‘dark’ state usually requires high illumination light intensity.

### Imaging cortical microtubule arrays in plant cells with VAEM-STORM imaging

**Figure 1:**
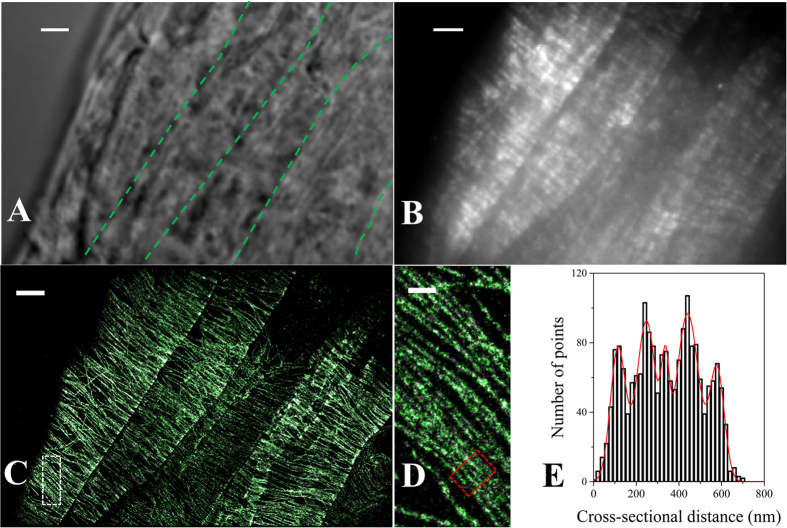
VAEM-STORM imaging of the cortical microtubule network of intact epidermal plant cells in the elongation zone of an Arabidopsis root tip. (**A**) Bright field image of cells at the elongation zone. The cells shown here are also highlighted in Fig. 4A as region d. The boundary between cells is highlighted with green dashed lines in the bright field image. (**B**) Epi-fluorescence image of the dense cortical microtubule network in the elongating cells and (**C**) the corresponding STORM image. (**D**) Zoomed-in STORM image as indicated in the white region in (**C**). (**E**) Cross-sectional profiles of a microtubule bundle in the cell [highlighted in the red box in (**D**)]. The histogram shows the cross-sectional distribution of the positions within the region specified by the red box. The red line is the fitting result from five simple Gaussian functions showing the centers of individual microtubules that are 80.0 nm ~ 137.9 nm apart. Scale bar: 3 μm (**A**–**C**) and 500 nm (**D**).

Images of the cortical microtubule arrays were reconstructed from 20,000–30,000 frames captured in 17–25 min. Due to the generally high background noise in imaging root samples, a slower frame rate of 20 Hz was chosen in the current study, when compared to several previously-published STORM imaging of animal cells^27^,^32^. To correct for the sample stage drift during the relatively long STORM image acquisition period, fluorescent beads were used as landmarks to track the stage movement and reconstruct the STORM images (e.g., Fig. 1C), which show higher spatial resolution compared to epi-fluorescence images (e.g., Fig. 1B and Supplemental Fig. 4A) and confocal microscopy images (Supplemental Fig. 4C,D). In the regions where cortical microtubules are present at high density and unresolved in epi-fluorescence and confocal microscopy images, individual microtubule filaments are clearly well-separated and resolved from each other in the STORM images. The complex organization patterns of cortical microtubule arrays in higher plants have diverse forms including random arrangement, regional organization, and eventually global organization. For example, parallel cortical microtubules in fast-elongating plant cells often co-align, forming microtubule bundles that are crucial for stabilizing the whole microtubule network. The bundles are believed to form by the crosslinking of individual microtubules with filamentous structures composed of microtubule-associated proteins^8^,^33^,^34^,^35^,^36^. Electron microscopy (EM) was used in these studies to reveal the details of cross-linking including the bridge-angle and inter-microtubule spacing in synthesized microtubule networks *in vitro*. As an example, in Fig. 1D,E, individual microtubule filaments in one bundle are clearly resolved, showing a spacing of 80–140 nm between neighboring microtubules, which is in agreement with the EM results^34^.

### Determination of the spatial resolution of VAEM-STORM imaging in plant tissues

**Figure 2:**
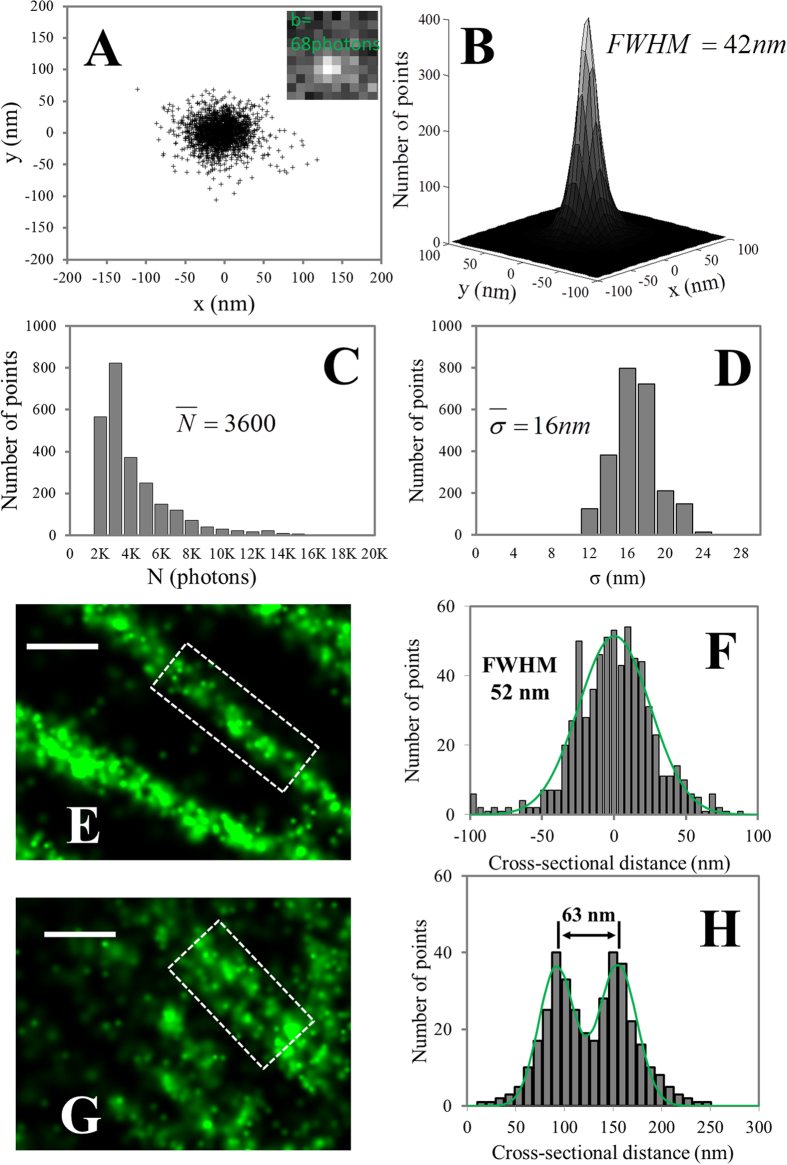
Estimation of the spatial resolution of VAEM-STORM imaging in intact Arabidopsis root tip cells. (**A**) Point-like objects appearing as clusters in the STORM image were used to directly determine the localization accuracy, thus estimating the spatial resolution. Inset shows a single molecule image in one frame. (**B**) The localization distributions of clusters. A histogram of the distributions of locations in 2D was generated by aligning about 30 clusters by their center of mass. The distribution was then fitted with a simple Gaussian function for determining the FWHM of 42 nm, which was used as the resolution for STORM imaging. (**C**) Photon numbers and (**D**) localization accuracies of Gaussian fitting of single molecule images from the same clusters as in (**B**). The typical image parameters, including the background noise b (68 ± 24 photons) and collected photons N from single molecules in their switching cycles (3,600 ± 1,297 photons), were determined. The average localization precision from a histogram of over 2,000 positions is 16.0 ± 2.2 nm. (**F**) The apparent width of a single free microtubule is ~50 nm from fitting the cross-sectional profiles of locations (white box, E, zoomed-in image are extracted from Fig. 1C) with a simple Gaussian function. The average apparent width of over 20 single free microtubules is 57 ± 4 nm (data not shown). The apparent microtubule width is a result of the actual size of the microtubule plus staining antibodies and the sub-50-nm imaging resolution. (**G**) VAEM-STORM imaging provides details of microtubule bundling structures in plant cells. (**H**) Microtubule bundles composed of two single microtubules (white region, G, zoomed-in image is extracted from Fig. 1C) with 62 nm separation are clearly resolved in STORM images. Space between individual microtubule filaments varies from 50–120 nm in animal cells and 20–40 nm in Arabidopsis cells depending on the crosslinking structures. The separation distance is larger as a result of broadening effects due to antibody sizes and the spatial resolution. Scale bar: 200 nm.

To quantify the achieved spatial resolution of VAEM-STORM imaging, point-like objects appearing as small clusters in the STORM images were used for analysis (Fig. 2A), as was described previously^4^,^37^. The lateral resolution was estimated to be 42 nm as the full width of half maximum (FWHM) from 2D Gaussian fitting of the distributions of locations (Fig. 2B). Alternatively, the resolution can be estimated from the localization accuracy^3^. The average localization precision (σ) is 16.0 ± 2.2 nm (Fig. 2D) from single molecule image analysis of the same clusters, giving a localization accuracy (~2.35σ) of 37.6 ± 5.2 nm. The results from both analyses agree well. Therefore, we have achieved a resolution of ~40 nm in intact plant tissue imaging.

We then measured the apparent width of individual cortical microtubule filaments in the STORM images by analyzing the cross-sectional profiles (Fig. 2E). Relatively long cortical microtubule filaments were chosen in order to obtain more reliable results. Fitting the cross-sectional distributions of the microtubule filament with a Gaussian distribution gives a FWHM of 52 nm (Fig. 2F). The average apparent width over 20 microtubule filaments is 57 ± 4 nm. The measured width is in agreement with the actual size of a microtubule filament plus staining with primary antibody and secondary F(ab’)_2_ fragments^38^. These results illustrate that the final spatial resolution depends both on the imaging method and the size of the objects. Therefore, microtubule filaments separated by ~60 nm should be readily resolved in VAEM-STORM imaging. As an example, two microtubules with 63-nm-separation are clearly resolved in Fig. 2G,H. Quantification of the ability to resolve nearby cortical microtubules based on Rayleigh criteria gives a resolution of 67 ± 8 nm (n = 15).

### Optimization of sample preparation and labeling

In order to label cytoskeletal structures in plant cells, permeabilization of the cell wall is required, as it presents an obstacle for the transfer of any probe larger than several nanometers into the cytoplasm. Several methods, including the digestion of cell walls with degradative enzymes (whole-mount)^39^, mechanical sectioning^40^ and freeze shattering combined with enzyme degradation^41^, have been previously reported. In addition, appropriate labeling density is critical for localization-based super-resolution imaging. The typical labeling concentrations reported in the literature were 1–10 μg/mL of dye-conjugated antibodies in animal cell imaging^4^,^27^. We attempted VAEM-STORM imaging of plant cells under such labeling conditions, using whole-mount samples in which the integrity of the cells is well preserved. However, due to high background and substantial interference between single molecule images resulting from the high labeling densities under such experimental conditions, no clear microtubule structures could be reconstructed (results not shown).

Alexa Fluor 647-conjugated F(ab’)_2_ fragments were chosen instead of whole IgG secondary antibodies in our experiments. F(ab’)_2_ fragments with a smaller size factor could penetrate the cell wall more easily than larger IgG secondary antibodies, thus offering better performance in both labeling and washing out free fragments. The dye-antibody ratio was ~4.5 for the dye-conjugated F(ab’)_2_ fragments we received from the manufacturer. To optimize the labeling density, we conducted two sets of experiments. In the first, combinations of different concentrations of both primary tubulin antibody and secondary F(ab’)_2_ fragment with whole mount immunostaining were used to label the cortical microtubules of Arabidopsis seedling roots (Supplemental Fig. 6A–D). The labeling concentration of Alexa Fluor 647-conjugated F(ab’)_2_ fragments was found to contribute the most to increasing the localization precision and thus the spatial resolution. The best spatial resolution of ~38 nm was achieved with 1.0 μg/mL primary antibody and 0.04 μg/mL secondary F(ab’)_2_ fragment, as a result of more photons being collected and lower background noise (Supplemental Fig. 6F–H). Continuous structures could not be completely reconstructed using more dilute labeling conditions (results not shown). These concentrations are ~50-fold lower than the typical concentrations used in the previously-published works^4^,^27^. The low concentration of antibodies reduces the labeling density, thus the number of activated probes during every activation cycle and increases the accuracy of point-localization during analysis^42^. Proper labeling density and relatively low background were achieved at these optimum antibody concentrations as demonstrated in Supplemental Fig. 6A–D. It is important to know that the best labeling concentrations vary according to the specific dye-conjugated secondary antibody used.

In the second set of experiments, we compared the whole mount method of sample preparation with the freeze shattering plus enzyme degradation method, in which intact roots were randomly split longitudinally, reducing the number of cell layers in the roots. Each method was followed with immunostaining of cortical microtubules using the optimized labeling concentrations. For both methods, sub-diffraction limited resolution was achieved for imaging cortical microtubules in Arabidopsis seedling root epidermal cells (Supplemental Fig. 6D,E). To quantitatively compare the spatial resolution achieved with these two sample preparation methods, cluster analysis was performed for each. The spatial resolution, estimated from the single molecule localization precision, was ~38 nm and ~23 nm for the whole mount and freeze shattering plus enzyme degradation methods respectively (Supplemental Fig. 6F). The increased resolution upon freeze shattering was primarily due to an increase in the number (*N*) of photons collected, from 3988 ± 53 photons up to 5525 ± 352 photons, and a reduction in *b* from 69 ± 8 photons to 54 ± 3 photons (Supplemental Fig. 6G,H). The increased photon counts and reduced background noise improve the localization accuracy by a factor of two, indicating that the multiple cell layers of intact roots is one important factor limiting the resolution achievable during super-resolution imaging in plant tissues. Especially in the case of the freeze shattering method, the spatial resolution was comparable to that achieved in animal cell imaging. Although it has been argued that minimal disruption of cellular components is introduced by freeze-shattering^41^, the continuous structure of the cortical microtubules was disrupted noticeably with this method as shown in the present work (Supplemental Fig. 6E). Therefore, we used the whole mount immunostaining method to label cortical microtubules with the photoswitchable dye molecule Alexa Fluor 647 for the remaining experiments.

### Quantitative analysis of the microtubule network with sub-50-nm resolution

The cortical microtubule array plays a key role in controlling plant cell growth and anisotropy through directly guiding the deposition of cell wall material. This occurs largely by determining the directional movement and plasma membrane insertion site of cellulose synthase complexes during cellulose synthesis and deposition, which constrains the direction of plant cell expansion^24^,^43^. Quantitative analysis of microtubule organization can therefore provide insight into the expansion and differentiation status of cells within the root^44^. Due to technical limitations, the details of microtubule structures have not been fully visualized or quantified. Using the enhanced spatial resolution of STORM images, quantitative data including microtubule density and orientation were obtained. Individual microtubules were resolved from one another even within crowded microtubule bundles, allowing the microtubule density to be accurately determined by simply counting microtubule filaments per micrometer. The uniformity of microtubule filament distribution within a cell is related to its differentiation status. Insights into cellular growth and differentiation could be acquired with accurate assessment of the microtubule density.

**Figure 3:**
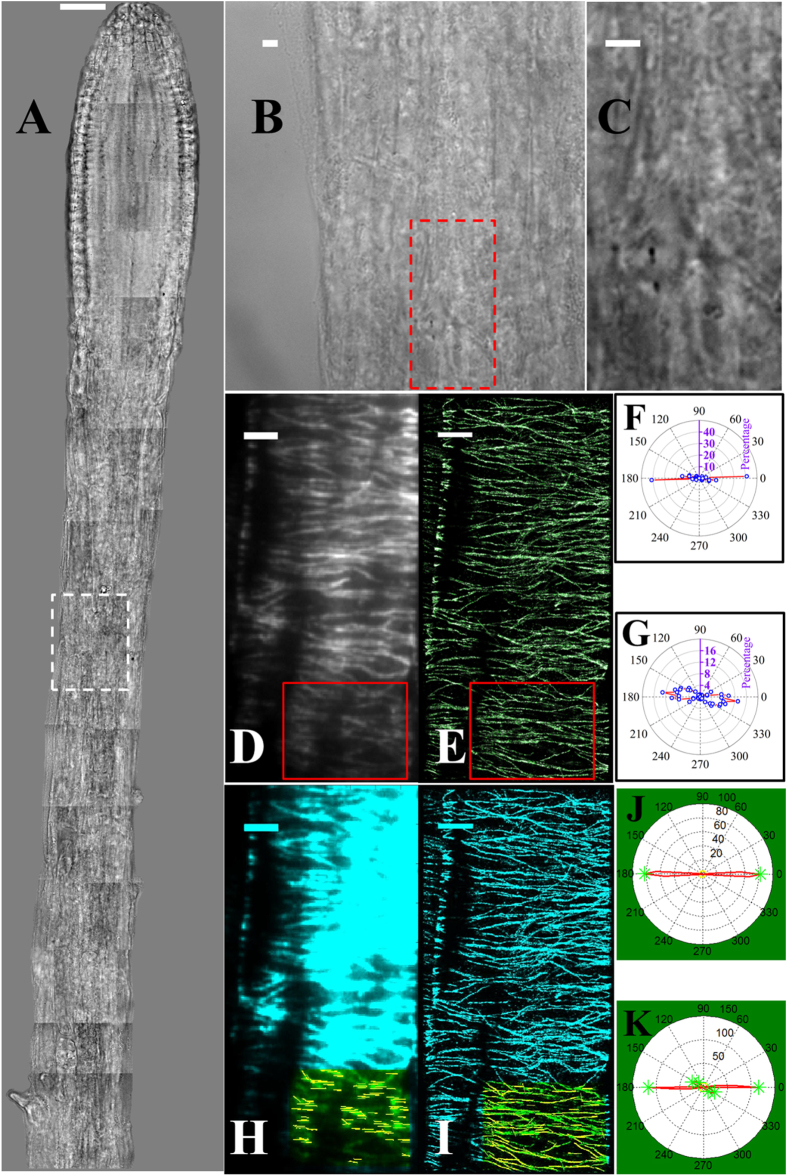
VAEM-STORM imaging of cortical microtubules in a plant epidermal cell of a whole intact root tip and orientation analysis. Analysis was performed both manually and using Microfilament Analyzer (MFA) software. (**A**) Bright field image of the whole root tip. Image is a collage of individual images with the same optical setup (100× objective) as for epi-fluorescence and STORM imaging. The location of cells for STORM imaging in the whole root therefore can be easily determined from the bright field images under the same magnification. (**B**,**C**) Zoomed-in bright field images showing the location of cells used for STORM imaging. (**D**) VAEM image and (**E**) STORM image of the cells highlighted in the bright field image, corresponding to the red box in (**B**). (**F**–**G**) Polar plots of the orientations of microtubule filaments in the VAEM image (**F**) and STORM image (**G**) as highlighted in the red boxes in (**D**,**E**), respectively. The long axis of the cell is indicated by the purple axis in the polar plots. (**H**–**K**) Results of orientation analysis using the MFA software. The offset angle is 90^0^ (long axis) and is not corrected in the polar plot, consistent with (**F**,**G**). Predominant angles are highlighted as green stars in the polar plot. Note that Figures (**F**,**G**) and Figures (**H**–**K**) are in different forms due to two different measuring methods. Scale bars: 30 μm (**A**) and 3 μm (**B**–**I**).

Although the predominant orientation of cortical microtubules can be identified from both epi-fluorescence and STORM images, much more detail is evident in the STORM images. Figure 3F,G show the distributions of cortical microtubules in the highlighted red boxes in the epi-fluorescence (Fig. 3D) and the STORM (Fig. 3E) image, respectively. In addition to the predominant transverse alignment, a significant population of microtubules aligning in between the transverse and longitudinal orientations can be resolved only in the STORM image. To verify the results from manual counting (Supplemental Fig. 7), MicroFilament Analyzer (MFA)^44^ was also used to analyze the angular distributions of cortical microtubules in both epi-fluorescence (Fig. 3H) and STORM (Fig. 3I) images. Consistently, more detailed structural information is found in the STORM images as demonstrated in the polar plots (Fig. 3J,K). It is therefore evident that STORM imaging, with the capability of resolving individual microtubules with sub-50-nm spatial resolution, helps in the accurate determination of the spatial organization, including density and orientation of cortical microtubule arrays, in plant cells.

### VAEM-STORM imaging of a whole Arabidopsis seedling root tip

The spatial organization of cortical microtubules can change as a response to the environment or during different developmental stages^45^,^46^. In Arabidopsis roots, the apical meristem is initially established during embryo development and provides stem cells for later, post-embryonic, growth. The defined division patterns of the stem cells lead to the formation of distinct developmental zones along the length of the root^47^,^48^. After cell division occurs in the meristem, cells leave the meristem and begin to elongate in a specialized zone termed the transition zone. Extensive rearrangement of the cytoskeleton occurs in the transition zone to allow the developmental switch to elongation. Cells then enter the elongation zone, in which rapid cell elongation occurs, and finally the more distal differentiation zone^49^.

**Figure 4:**
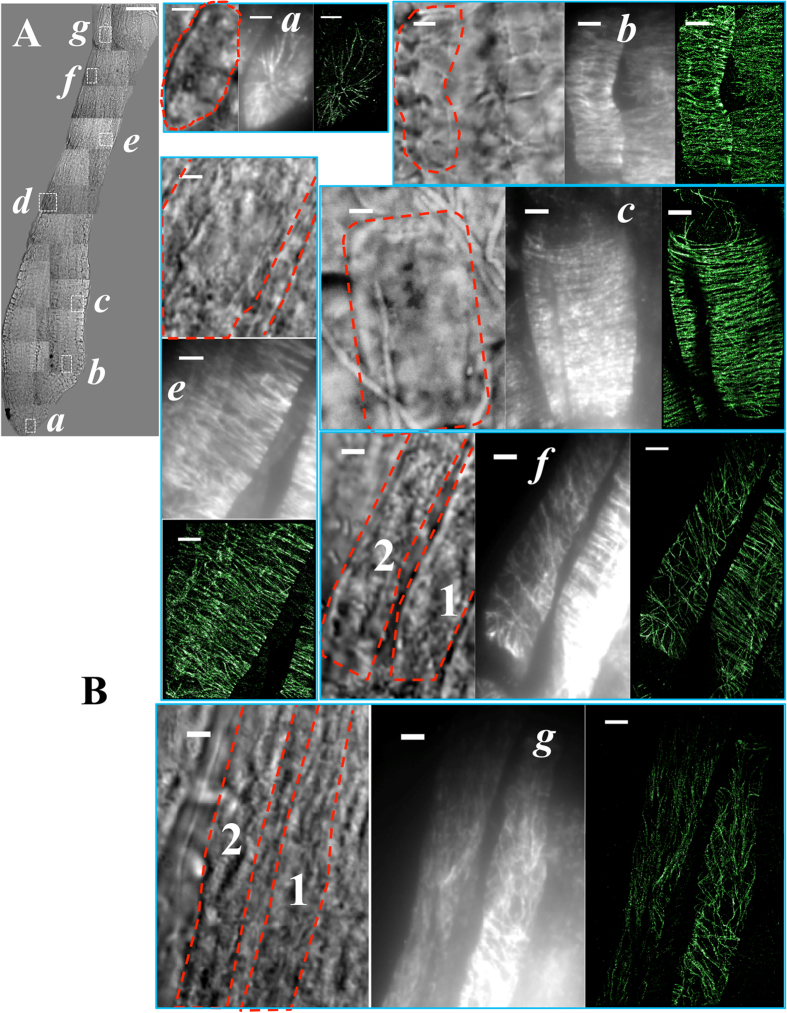
VAEM-STORM imaging of cortical microtubules in different parts of an intact Arabidopsis root. (**A**) Mosaic of bright field images of the root tip. Seven regions (***a-g***) across the root tip are highlighted and were chosen for dSTORM imaging. (**B**) Zoomed-in bright field and corresponding epi-fluorescence and STORM images of the white-boxed regions in (**A**). Images are shown of cortical microtubules of cap cells (***a***), epidermal cells in meristematic zone (***b***), elongation zone (***c-d***), differentiation zone (***g***) and transition zones (***e-f***) between elongation and differentiation of the intact Arabidopsis root tip. Note that the zoomed-in bright field image, epi-fluorescence image and STORM image of cells in region ***d***are shown in Fig. 1. Cell boundaries are highlighted with red dashed lines. Cells in regions ***f***and ***g*** show distinct microtubule organization patterns, thus they are analyzed separately in Fig. 5 as ***f***_***1***_, ***f***_***2***_ and ***g***_***1***_, ***g***_***2***_ as indicated in the corresponding bright field images. Scale bars: 50 μm (**A**) and 3 μm (**B**, ***a-g***).

Seven regions (***a-g***) spread throughout a root tip, as highlighted in the white boxes in Fig. 4A, were chosen for STORM imaging. Region ***a*** is within the apical cap; ***b*** is in the meristematic zone; ***c-d*** are in the elongation zone; ***e-f*** are between the elongation zone and the differentiation zone; and finally, ***g*** is in the differentiation zone. Variations in density and orientation of microtubule arrays between different zones in the root tip can be observed qualitatively in both epi-fluorescence images and STORM images; however, accurate quantitative structural information, which varies dramatically in different zones, is only available in STORM images (Fig. 4B).

The STORM images show that cortical microtubules in rapidly elongating cells (Figs 1C and 4B *c*) are preferentially present in bundled structures, while single unbundled microtubules are commonly found in the apical cap cells and meristematic cells (Fig. 4B a,b) and the differentiated cells (Fig. 4B g) where the microtubules are less organized and at lower density. Moreover, the transition regions (Fig. 4B e,f) between different zones have intermediate microtubule density, indicating the changing organization during different growth phases. It has been suggested that the highly spatially organized and more stable regions of the cortical microtubule arrays are formed from bundled microtubules; the bundling lends stability to the overall structure despite the rapid treadmilling behavior of individual filaments^50^. In contrast, the less organized and short-lived areas were hypothesized to correspond to individual microtubules present at lower density that move and reorganize via treadmilling^51^. To provide direct evidence for this hypothesis, super-resolution is necessary to distinguish individual microtubules within bundles and to accurately assess microtubule density in different regions of the plant root.

**Figure 5:**
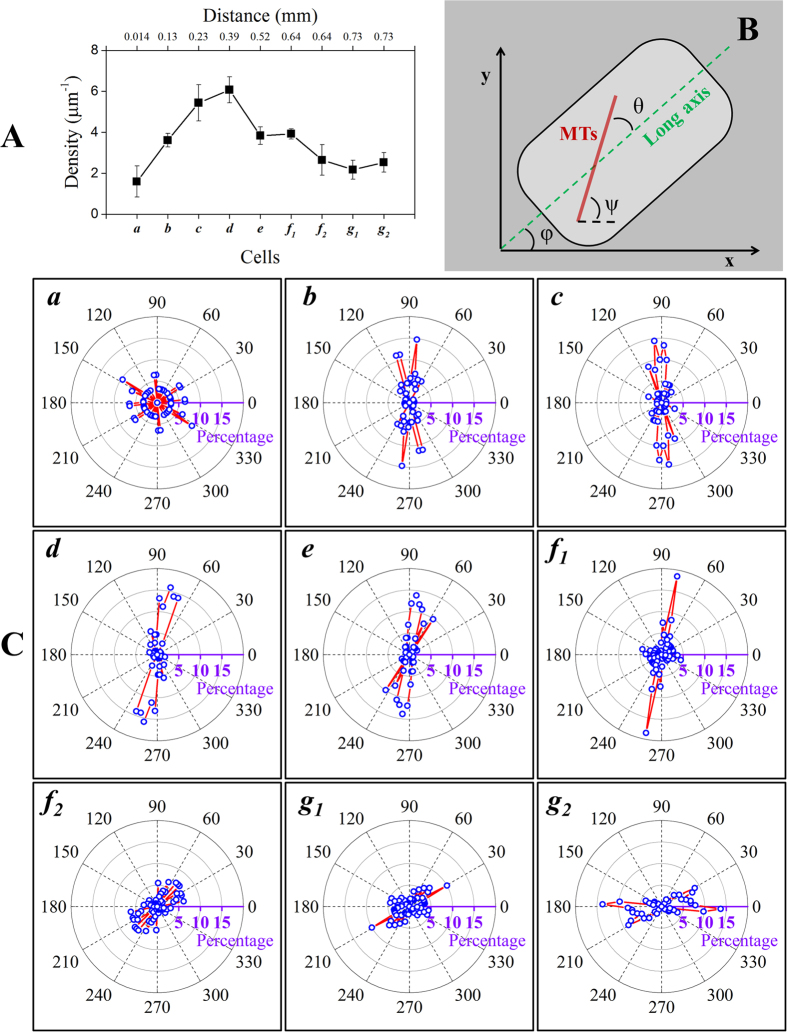
Quantitative analysis of the cortical microtubule network in a plant root tip. (**A**) The average cortical microtubule density (number of microtubules per micrometer) in the highlighted cells in Fig. 4, with the distance of cells from the root tip. Error bars represent the standard deviation of densities of cortical microtubules from over 10 sub-regions in each cell. (**B**) Schematic representation of the orientation of cortical microtubules, taken as the relative angle (θ) of each microtubule filament to the long axis of the cell. The orientation angle θ as displayed in (**C**) is corrected by the offset angle φ of the cell, determined by its long axis. Ψ is the angle of the microtubule filament in the coordinates of the STORM image (x–y). All angles were determined by using NIH ImageJ (http://rsbweb.nih.gov/ij/). (**C**) Polar plots showing the distributions of cortical microtubule orientations (θ) of the corresponding cells in Fig. 4. Adjacent cells (***f***_***1***_ and ***f***_***2***_, ***g***_***1***_ and ***g***_***2***_) showing dramatically different cortical microtubule organizations were analyzed separately. Note that the long axis of the cell has been aligned to the 3 o’clock position in all polar plots as highlighted in purple.

Figure 5 shows the results from the quantitative analysis of the density and orientation of cortical microtubules in the cells highlighted in Fig. 4. Statistics provided in Supplemental Table 1 indicate that the microtubule densities are not significantly different (P > 0.1) between regions ***c-d***, ***e-f***_***1***_, ***f***_***2***_***-g***. Root cap cells (***a***) have loosely organized cortical microtubule structures at low density (1.6 ± 0.8 μm^−1^) and rarely form microtubule bundles. Cells in the late meristematic zone (***b***) have an increased microtubule density, and the cortical microtubules are arranged more tightly in the transverse direction. In the elongation zone (***c, d***), cortical microtubules strongly prefer to exist as bundles in the rapidly growing cells, and as expected, the density is 3–4 times higher (~6.0 μm^−1^) than in region ***a***. For the cells in the late elongation or early differentiation stages (***e, f***_***1***_), the microtubule density decreases to ~4.0 μm^−1^. Cortical microtubule structure becomes loose (~2.0–2.5 μm^−1^) and preferentially exists as free microtubule filaments in the differentiation zone (***f***_***2***_***, g***), which is consistent with decreasing growth.

Microtubule orientation, on the other hand, varies dramatically in different zones of the root tip. This set of data shows that microtubule orientation changes from random alignment at low density (***a***), to transverse alignment (90° to the long axis of the cell) at high density (***b***-***f***_***1***_), then to random alignment again at intermediate density (***f***_***2***_-***g***_***1***_), and finally to longitudinal alignment at low density (***g***_***2***_). Consistent with previous reports^52^, cortical microtubules are more likely to have a random organization in the root apical area (***a***) where cells are in the process of division, and no predominant orientations are found in this region. A transverse alignment is predominant in ***b***to ***f***_***1***_ with only small variations among these regions. Nonetheless, a significant population of microtubules with small orientation angles are present in ***f***_***1***_ which does not exist in ***b-e***. In contrast to ***f***_***1***_, the adjacent cell ***f***_***2***_ has no predominant orientations (with angles spanning the full range), which is consistent with the helical structure of cortical microtubules^39^ in ***f***_***2***_. The variability in microtubule orientation continues to increase in ***g***_***1***_; however, its adjacent cell ***g***_***2***_ shows a predominant orientation angle parallel to the cell’s long axis. The difference in microtubule organization at the same distance from the root tip (***f***_***1***_ vs. ***f***_***2***_and ***g***_***1***_ vs. ***g***_***2***_) could be because neighboring cells are at different differentiation stages or, alternatively, because they are of different cell types (epidermal and cortical cells)^22^.

## Conclusions

In conclusion, we have successfully applied direct STORM imaging to cortical microtubules in Arabidopsis root cells with sub-50-nm resolution. The optimized labeling strategy, with much lower antibody/fragment concentrations compared to those reported in the literature, was critical for achieving such high resolution in plant cell and whole tissue imaging. Different sample preparation methods, including the commonly used enzymatic degradation of the cell wall and a combination of freeze-shattering and enzymatic cell wall degradation, were optimized to provide a spatial resolution of up to 20 nm, which is close to the best resolution demonstrated in mammalian cells. Finally, we quantified the spatial organization of cortical microtubules in different parts of an intact Arabidopsis root tip. The quantitative results show the dramatic differences in the density and spatial organization of cortical microtubules in cells of different differentiation stages or types. The methods developed can be applied to a wide range of plant cell biological processes, including imaging of additional elements of the cytoskeleton, organelle substructure, membrane domains and other structures currently only accessible by electron microscopy. We expect these advances in super-resolution imaging will enable critical progress in understanding the detailed spatial organization of biological processes in plant cells.

## Methods

### Optical setup for VAEM-STORM

The imaging system for VAEM-STORM (Supplemental Fig. 1) was integrated into an inverted microscope (Zeiss Axiovert 100 TV, Jena, Germany). Multicolor lasers were collimated into a single light path after the beam expander (Thorlabs BE03M-A, Newton, NJ) with 3 × magnification. Illumination light was provided by solid state lasers operating at different wavelengths (Newport Excelsior one 405 nm, 200 mW, Irvine, CA; Laser Quantum Gem 660, 200 mW, Stockport, Cheshire, England). Collimation of multicolor lasers was accomplished by using a dichroic mirror (Thorlabs, DMLP425T), thus allowing simultaneous illumination of the sample at multi-wavelengths. Uniblitz mechanical shutters (Vincent Associates, LS2Z2, Rochester, NY) in front of each laser were used to control the illumination conditions, either pulsed or continuous illumination profiles. The collimated light was expanded by a telescope of a pair of achromatic lenses (Thorlabs, AC127–025-A & AC254–150-A) and then focused at the back focal plane of a high refractive index oil immersion objective (Nikon, 100X Oil, N.A. 1.49) using another achromatic lens (Thorlabs, AC508–200-A). The incident angle of illumination light is controlled by the lateral shift of the light path, through a three-dimensional stage (Sigma KOKI, SGSP-20-20, Tokyo, Japan), before entering the objective. A multi-edge beam splitter (Semrock, DC-405-388-543-635, Rochester, NY) was used to reflect the light into the working objective to excite the sample and the emission light is collected by the same objective. After the tube lens provided with the microscope, a pair of relay lenses (Thorlabs, AC127–125-A) was used to focus emission light onto an EMCCD chip (Andor iXon^EM^ + 897) enabling a pixel size of ~160 nm. A combination of filters (Semrock, 664 nm RazorEdge long-pass edge filter (LP02-664RU-25), 658 nm StopLine single-notch filter (NF03-658E-25), 708/75 nm BrightLine single-band bandpass filter (FF01-708/75-25)) was inserted in front of the camera to reduce the background noise. Epi-fluorescence imaging under variable angle illumination and VAEM-STORM imaging were performed using the customized system. Confocal images were acquired with a Leica confocal laser scanning microscope (Leica Microsystems, Leica SP5, Buffalo Grove, IL) using a × 63 Leica oil-immersion objective. Excitation and emission wavelengths were 652 and 668 nm respectively.

### Plant materials and growth conditions

*Arabidopsis thaliana* Columbia-0 seeds were surface sterilized with 33% bleach (Clorox, Oakland, CA), 0.1% (v/v) Triton X-100 (Sigma, St. Louis, MO) for 20 min, followed by 5 rinses with deionized water. Sterilized seeds were kept in the dark at 4 °C for 2 days. Arabidopsis seedlings were grown vertically for 3–5 days under long-day conditions (16 hours light) on half-strength MS agar medium (Murashige-Skoog vitamin and salt mixture, MSPA0910, Caisson, Colorado Springs, CO) with 1% sucrose (Sigma), 2.4 mM pH 5.7 MES (Sigma), and 0.6% (w/v) Phytoblend (Caisson).

### Immunofluorescence staining by whole mounting

Arabidopsis seedlings were immunostained as previously described^39^ with some modifications. Three- to five-day-old Arabidopsis seedlings were fixed for 40 min in fixation solution (1.5% paraformaldehyde (Sigma) and 0.5% glutaraldehyde (Sigma) in PME buffer [50 mM PIPES (J.T. Baker, Center Valley, PA), 2 mM EGTA (Sigma), 2 mM MgSO4 (Fisher)] with 0.05% Triton X-100). The fixed samples were washed 3 times (10 min each time) with PME buffer followed by digestion for 20 min with 0.05% (w/v) Pectolyase (Karlan, Cottonwood, AZ) in 0.4 M mannitol (Sigma) in PME buffer. The samples were washed again in PME buffer for 3 × 5 min. The samples were treated with –20 °C methanol (Fisher) for 10 min and rehydrated in phosphate-buffered saline (PBS) buffer for 10 min. 1 mg/mL NaBH_4_ (Fisher) in PBS was applied to the samples for 20 min to reduce autofluorescence and then 3% BSA (Fisher) in PBS supplemented with 50 mM Gly (MP Biomedicals) for 3 hours for blocking. The samples were incubated with mouse anti-α-tubulin primary antibody (Sigma, T6074) at 1:1000–1:2000 dilution in 1% BSA in PBS at 4 °C overnight. Samples were washed three times for 10 min each and incubated in Alexa Fluor 647-conjugated F(ab’)_2_ fragment of goat anti-mouse IgG (H + L) (Invitrogen, A-21237) at 1:20,000–1:50,000 dilution in 1% BSA in PBS for 3 hours at 37 °C. After rinsing 5 times (10 min each time) in PBS, samples were post-fixed with fixation solution, washed a further 3 times (10 min each time), and mounted on a coverslip in imaging buffer [100 mM Tris pH 8.0 (Ambion), 10 mM NaCl (Sigma), 0.5 mg/mL glucose oxidase (Sigma), 40 μg/mL catalase (Sigma), 10% (w/v) glucose (Sigma) and 1% (v/v) β-mercaptoethanol (Sigma)] for epi-fluorescence/STORM imaging or kept in PBS buffer at 4 °C for up to one month.

### Immunofluorescence staining by freeze shattering

Freeze-shattering was performed as described previously^41^ with modifications. Three- to five-day-old Arabidopsis seedlings were fixed in fixation solution, washed 5 times with PME buffer and digested with enzymes (identical to the procedure described in the previous section for the whole mounting method). After digestion, samples were placed between two 3-Aminopropyltriethoxysilane (APTES) coated slides and frozen immediately in liquid nitrogen for 5 min. After removal from liquid nitrogen, the paired-slides were immediately separated and air-dried for 40 min. Freeze-shattered samples were permeabilized on the slides using 0.5% [v/v] NP40 (USB) and 10% DMSO (Fisher) in PME buffer and washed three times with PME buffer (10 min each time). The samples were reduced using 1 mg/mL NaBH_4_ in PBS for 20 min and blocked with 3% BSA in PBS for 1.5–2 hours. Primary antibody and secondary fragment were as above. The samples were post-fixed for 20 min in fixation solution. For long-time storage, samples were immersed in PBS buffer and kept at 4 °C for up to one month.

### Sample preparation and drift correction using fiducial marker

For intact root samples, the labeled root was placed on a 22 mm × 50 mm Corning No.1 coverslip (Corning, NY). For the freeze-shattering procedure, glass slides coated with APTES were used to attach the root to the surface. Imaging buffer was applied to the sample slide and then an 18 mm × 18 mm Corning No.1 coverslip was applied on the sample to form a chamber and sealed using nail polish to avoid oxygen from the air, thus allowing imaging for several hours. The sample chamber was then placed on a customized sample holder and was locked with two metal clips to reduce the sample drift. The sample holder was mounted on a high performance three-dimensional piezo stage (Physik Instrumente, P-527.3CD), which was integrated into the microscope system. Fluorescent beads (Invitrogen, T7279, Carlsbad, CA) absorbed on the coverslip or glass slides were used as fiducial markers. The positions of the beads were tracked and used for stage drift correction of localized molecular positions in STORM images.

### Localizing the center of single molecules and determining localization accuracy

The molecular size of single fluorescent molecules is usually a few nanometers; however the corresponding microscopic images have a typical size of a few hundred nanometers because of the point spread function (PSF) of the imaging system. Though the true molecular size of molecules cannot be determined using fluorescence microscopy, the centroid of the molecule can be localized with high precision with fitting by its PSF or 2D Gaussian function as an estimate. The precision of the localization, in brief, is inversely proportional to the square root of photons (√N) collected from the single molecule^8^. Molecules emitting several thousand photons can be localized with nanometer precision under this standard. The simple estimation model works well in cases where the background noise is very small so that it can be neglected. In circumstances such as plant cell imaging, background noise is often high. Localization precision is measured by using another equation according to Thompson et al.^53^.


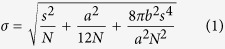


where *s* is the standard deviation of the Gaussian distribution that equals 1/2.2 of the PSF width, *a* is the pixel size, *b* is background noise and *N* is number of collected photons. The first term (*s*^2^/*N*) is the photon noise, the second term is the effect of finite pixel size of the detector, and the last term is the effect of background. Using point-like objects appearing as clusters in the STORM images, the fitting parameters (Fig. 2A) were extracted *b*: 68, *N*: 3600, *a*: 156 nm giving an average localization of 16 nm. The FWHM of the fitting is used, giving an estimation of the spatial resolution as 40 nm. The spatial distributions of the clusters were also fitted by 2D Gaussian function giving a FWHM of 42 nm (Fig. 2B), in agreement with the results from single molecule fitting. The spatial resolution in VAEM-STORM imaging of intact Arabidopsis root cells in our experiments therefore was comparable to the resolution in animal cell imaging.

### FWHM from distributions of locations suggest the spatial resolution

2D Gaussian fitting of the distributions of position clusters in STORM images had been demonstrated previously to estimate the actual spatial resolution^3^,^4^. We hereby use simulations with parameters extracted from our experimental data to better illustrate this method. Clusters with an uncertainty of ~17 nm (same as our experimental data) for point sources are generated in MATLAB. Each cluster contains 200 random positions. Supplemental Fig. 5a shows two clusters that are well separated by a distance of 100 nm between their centers. When the distance between two clusters decreases to be exactly the FWHM (42 nm), the two clusters are still clearly separated (Supplemental Fig. 5b). Under this separation distance, the cross-sectional profiles of the two clusters interact at the place where the intensity drops to 50% of their peak intensity (Supplemental Fig. 5b, right panel). When the distance between two clusters decreases even further (such as 20 nm), the two clusters merge into one (Supplemental Fig. 5c). These simulated results clearly demonstrate the ability of this method for estimating the actual spatial resolution for a given super-resolution image.

### Data analysis of VAEM-STORM imaging

VAEM-STORM imaging data was processed using Insight3 software kindly provided by Dr. Bo Huang, University of California at San Francisco. In our experiments, an imaging sequence of 20,000–30,000 frames recorded at 20–60 Hz was used to reconstruct a high resolution STORM image. In each frame, individual molecules were identified and fit by an elliptical Gaussian function to determine their centroid positions, widths, intensities and ellipticities. A threshold was chosen to eliminate molecules that were too dim, too wide or too elliptical to yield high localization accuracy based on the fit parameters. Usually, the intensity of the illumination light and the recording speed was adjusted such that fluorescent molecules were in the ‘on’ state for 2–3 frames. Molecules appearing in consecutive frames with a displacement smaller than one pixel were considered to be from the same fluorescent molecule and their final positions were determined using the weighted centroid positions in all consecutive frames.

A pixel size of 10 nm was used to generate the STORM images. In the STORM images, each molecular position was assigned as one point. These points were rendered as a normalized 2D Gaussian peak, the width of which was determined by its theoretical localization accuracy calculated from the number of photons detected for that localization event.

## Additional Information

**How to cite this article**: Dong, B. *et al.* Stochastic Optical Reconstruction Microscopy Imaging of Microtubule Arrays in Intact Arabidopsis thaliana Seedling Roots. *Sci. Rep.* **5**, 15694; doi: 10.1038/srep15694 (2015).

## Supplementary Material

Supplementary Information
