# Supplementary material for: Stochastic Optical Reconstruction Microscopy Imaging of Microtubule Arrays in Intact Arabidopsis thaliana Seedling Roots
Source: Sci Rep. 2015 Oct 27;5:15694. doi: 10.1038/srep15694 (PMC4621606; doi:10.1038/srep15694)
Supplement: Supplementary Information [file srep15694-s1.pdf]

Supplementary Information

Stochastic Optical Reconstruction Microscopy Imaging of Microtubule Arrays in Intact *Arabidopsis thaliana* Seedling Roots

Bin Dong[1,§], Xiaochen Yang[2,§], Shaobin Zhu[1], Diane C. Bassham[2,3,*], and Ning Fang[4,*]

1. Ames Laboratory, U.S. Department of Energy and Department of Chemistry, Iowa State University, Ames, Iowa 50011
2. Department of Genetics, Development and Cell Biology, Iowa State University, Ames, Iowa 50011
3. Plant Sciences Institute, Iowa State University, Ames, Iowa 50011
4. Department of Chemistry, Georgia State University, P.O. Box 3965, Atlanta, Georgia 30302

§These authors contributed equally to this work.

***Authors for correspondence:**

Diane C. Bassham, bassham@iastate.edu

Ning Fang, nfang@gsu.edu

Supplemental Figure 1. Schematic diagram of the setup for VAEM-STORM imaging.

Supplemental Figure 2. Comparison of laser beam paths.

Supplemental Figure 3. Comparison of S/N between epi-fluorescence illumination and VAE illumination.

Supplemental Figure 4. Diffraction unlimited resolution in VAEM-STORM imaging compared to diffraction limited imaging of cortical microtubules in plant cells in the elongation zone.

Supplemental Figure 5. Simulation data showing the resolution of two separated clusters.

Supplemental Figure 6. Proper labeling density of cortical microtubules by antibody-conjugated dye molecules is critical for generating super resolution images with sub-50nm-spatial resolution.

Supplemental Figure 7. Quantitative information on the cortical microtubule network is readily available with a resolution of 20 – 40 nm in STORM images.

Supplemental Table 1. T-test of densities of cortical microtubules between adjacent regions (***a*-*g***) highlighted in Fig. 4.

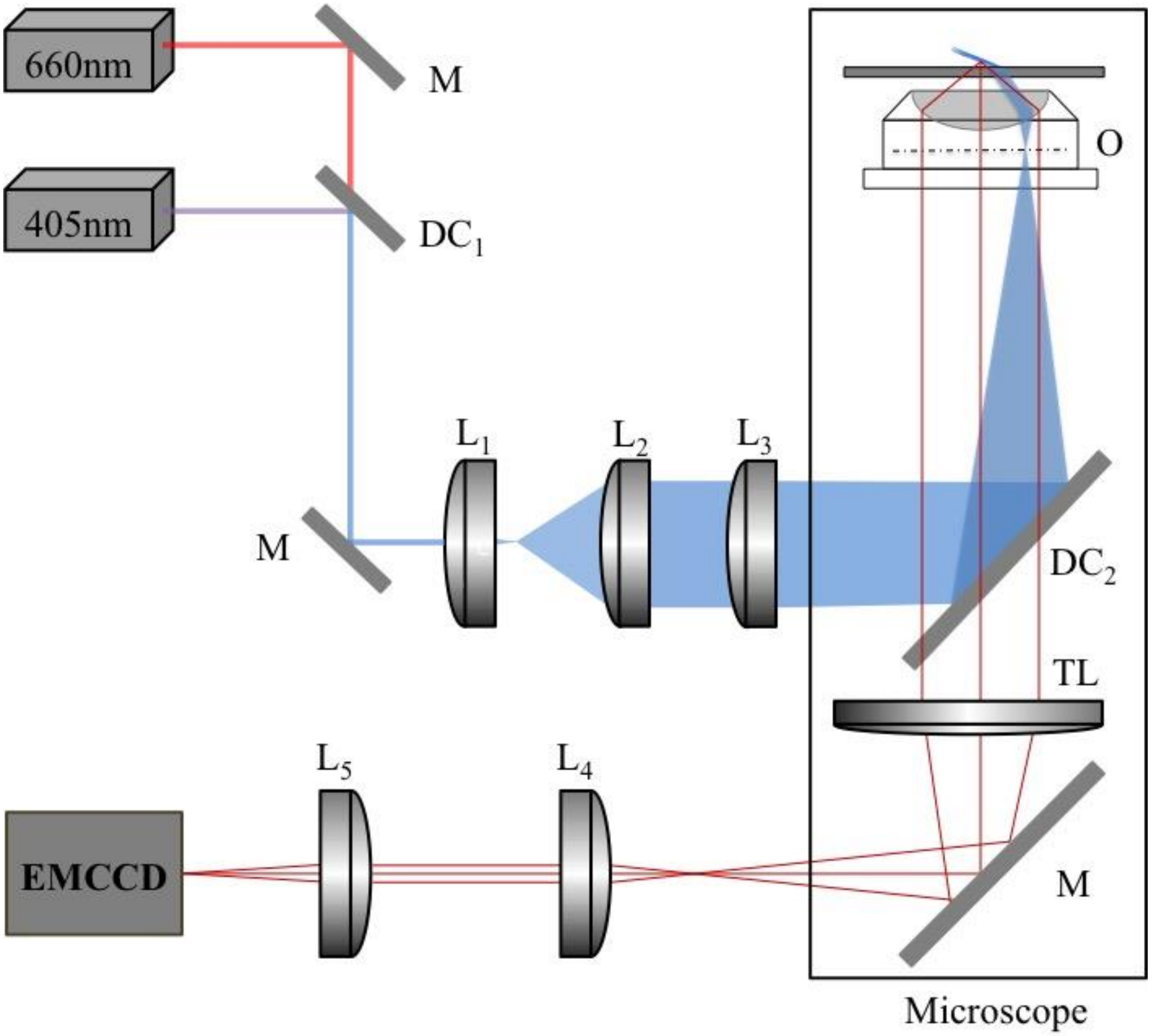

Supplemental Figure 1. Schematic diagram of the setup for VAEM-STORM imaging. Multiple lasers were collimated by using dichroic mirrors DC_1. Collimated lasers were first expanded with two achromatic lenses L_1 (Thorlabs, AC127-025-A) and L_2 (Thorlabs, AC254-150-A) and then focused by an achromatic lens L_3 (Thorlabs, AC508-200-A) at the back focal plane of an oil objective O (N.A. = 1.49). Dotted-dashed and solid lines represent the back focal plane of O and the sample-imaging plane respectively. The single molecule images generated by the objective and tube lens (TL) were imaged by an Andor iXon+ 897 EMCCD camera via a pair of relay lenses L_4 and L_5 (Thorlabs, AC254-125-A). Emission filters are inserted between L_4 and L_5 to exclude scattering background. A highly inclined illumination condition was achieved via lateral shifting of the position of L_3, which was mounted on a motorized 3D stage.

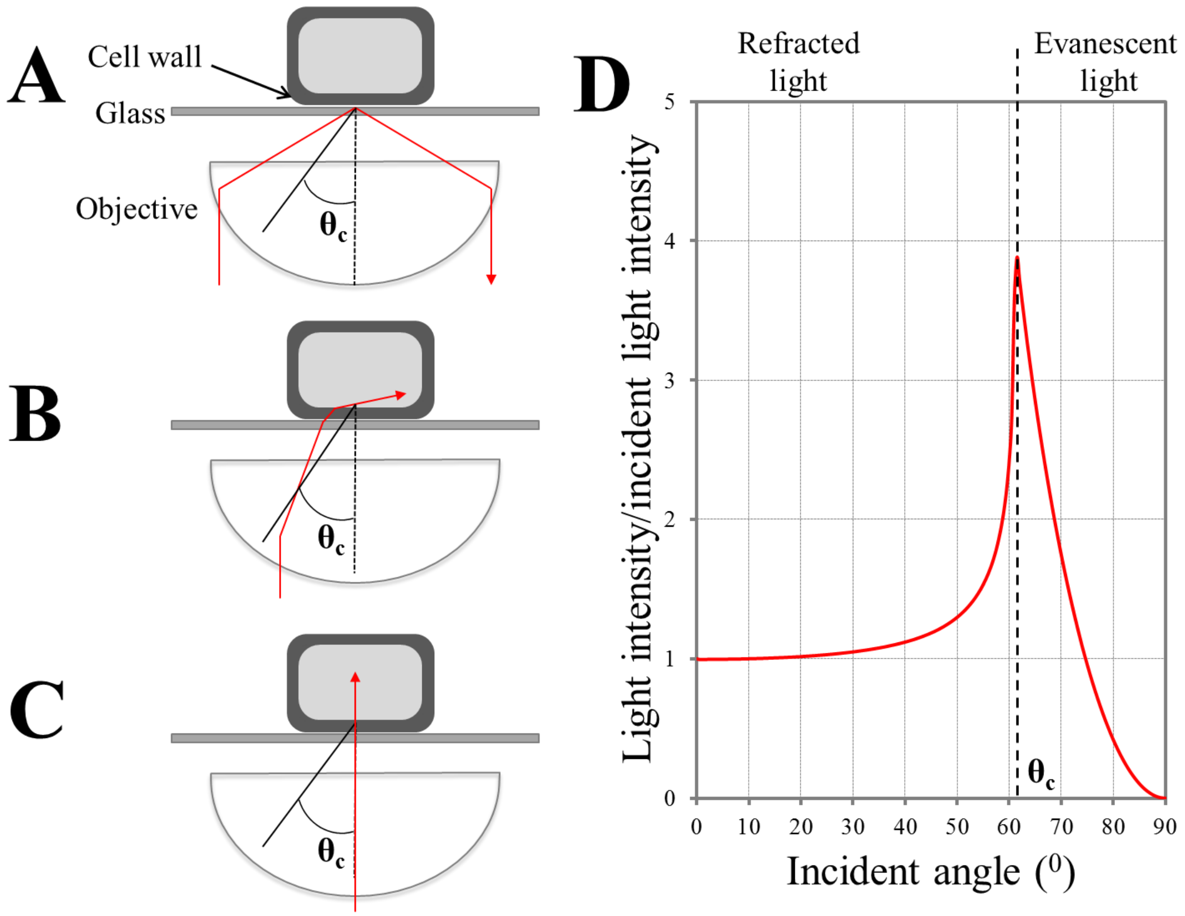

Supplemental Figure 2. Comparison of laser beam paths. Comparison of the laser beam paths of total internal reflection fluorescence (TIRF) (A), variable angle epi-fluorescence (VAE) (B) and epi-fluorescence (C) illumination. In VAE geometry, the incident angle of the laser beam is slightly smaller than yet very close to the critical angle. The illumination depth is adjusted by scanning the subcritical angles to achieve the illumination condition giving the best signal to noise ratio. (D) Theoretical light intensity on the coverslip-specimen surface at the specimen side, when a beam is introduced from the glass side, as a function of the incident angle. At $\theta < \theta_c$, the laser beam is refracted. At $\theta \geq \theta_c$, the beam is totally internally reflected. Within about 10° from the critical angle, the light intensity on the specimen side is larger than that of the incident laser beam. When the incident angle is increased up to the critical angle, the intensity of refracted light increases due to the reduced thickness of the refracted beam at large incident angles. This provides the necessary pre-condition for tuning the switching properties of dye molecules. At the critical angle, the laser intensity of the evanescent field is about four times higher than the incident beam. This is because the light intensity at the surface is the sum of the incident and

reflected beams whose phases are the same. Therefore, the electric field of the total light doubles while the light intensity increases by a factor of four, which is the square of the electric field. As the incident angles become larger than the critical angle, the phases of the incident and reflected beams are different, thus the light intensity of the evanescent field decreases.

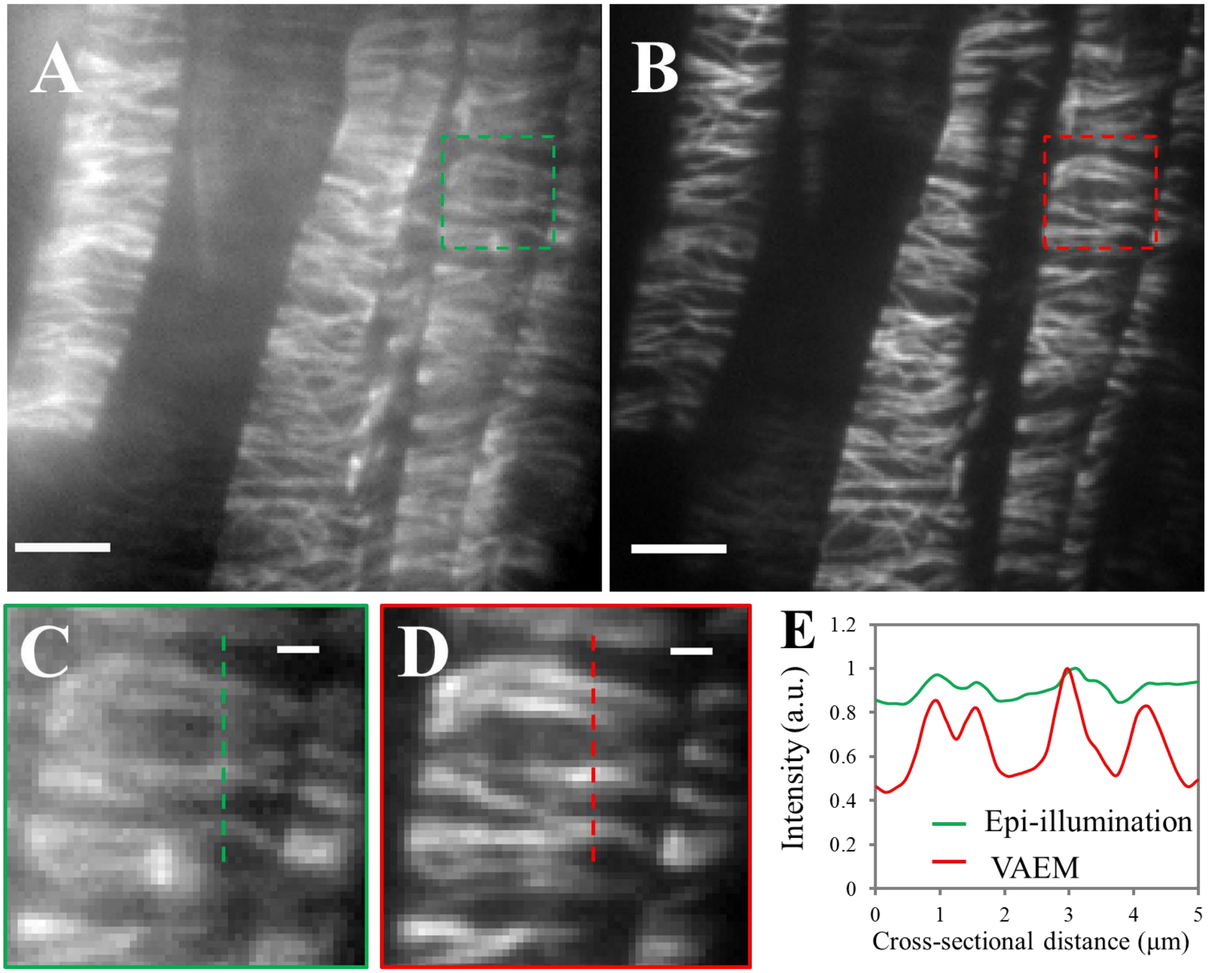

Supplemental Figure 3. Comparison of S/N between epi-fluorescence illumination and VAE illumination. Imaging of Alexa Fluor 647-labeled cortical microtubules under epi-fluorescence (A) or VAEM (B). Images are acquired for epi-fluorescence and VAEM imaging using the same excitation intensity at 660 nm (3.5 kW/cm2) and same exposure time (50ms). The corresponding close-up images (C, D) show that the background due to out of focus contributions in VAEM is greatly reduced compared to epi-fluorescence. (E) Cross-sectional profiles at the same location show the dramatically enhanced S/N in VAEM compared to epi-fluorescence. Scale bar: 5 μm (A, B) and 1 μm (C, D).

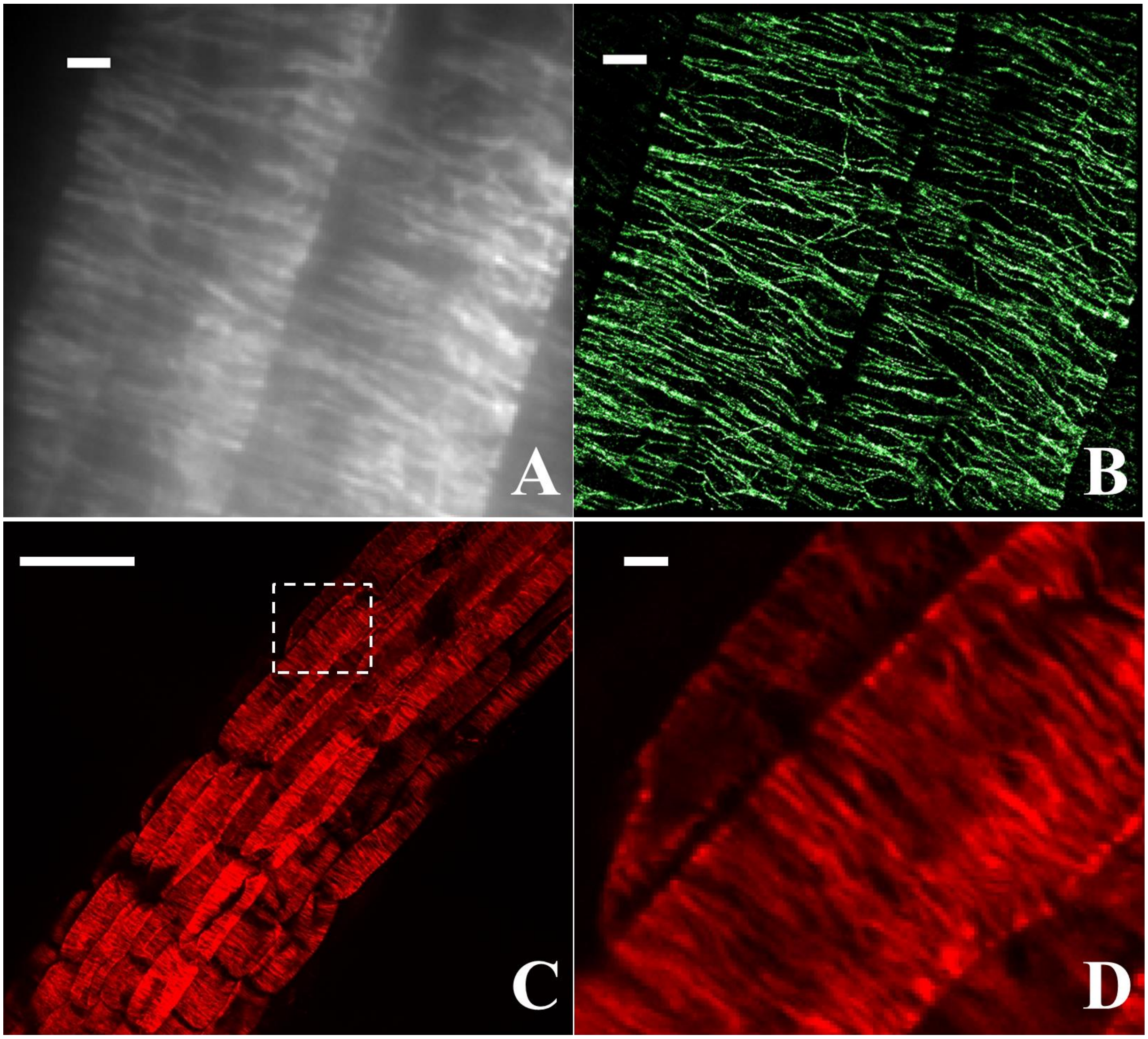

Supplemental Figure 4. Diffraction unlimited resolution in VAEM-STORM imaging compared to diffraction limited imaging of cortical microtubules in plant cells in the elongation zone. (A) Epi-fluorescence image and (B) STORM image of the same area. (C) Confocal image and (D) zoomed-in image showing the highly packed cortical microtubules in plant cells. Compared to the images taken by epi-fluorescence microscopy and confocal microscopy, where close cortical microtubules are not resolvable, the structure and organization of the cortical microtubules in the STORM images are much clearer and better resolved. Scale bar: 2 µm (A, B, D) and 50 µm (C).

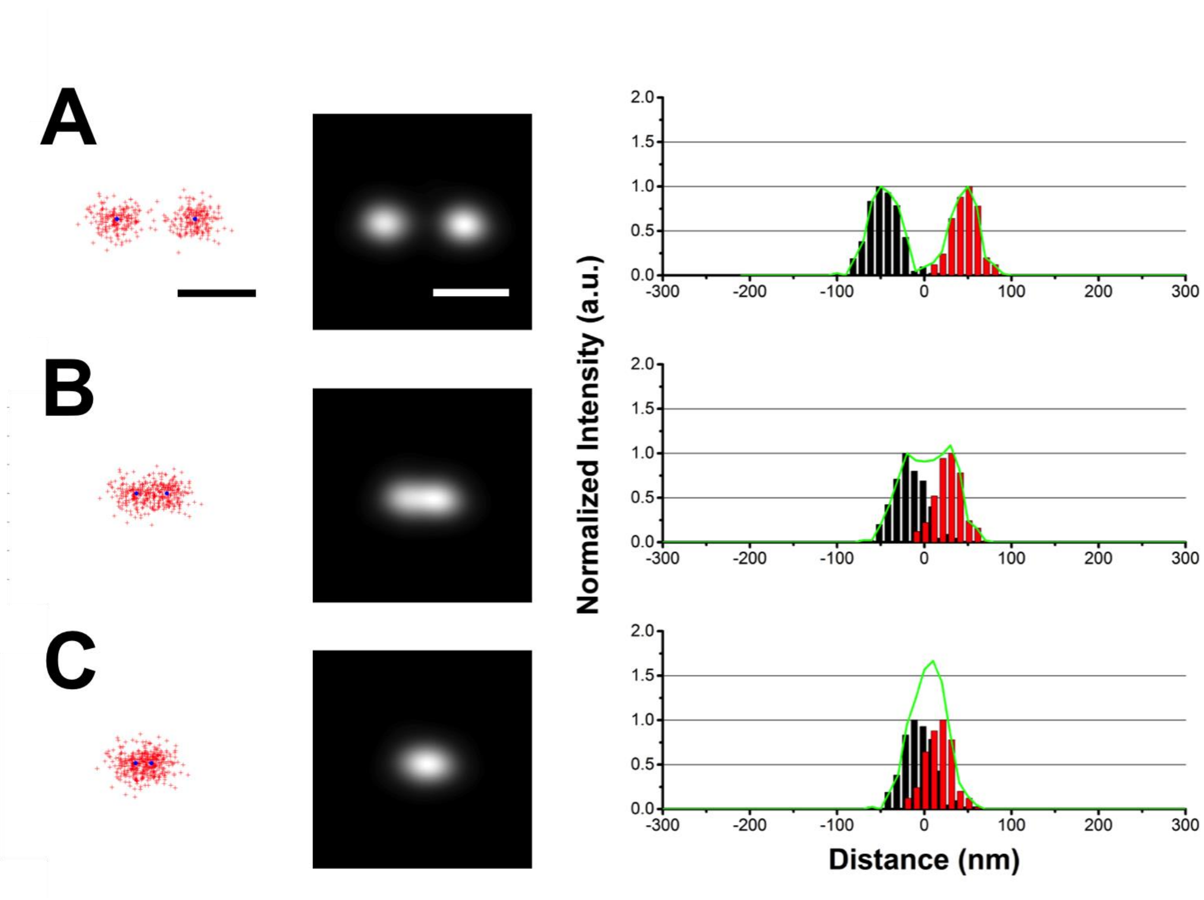

Supplemental Figure 5. Simulation data showing the resolution of two separated clusters. The two clusters are separated by distances of 100 nm (A), 42 nm (B), and 20 nm (C), respectively, in the left panel of the figure. Each cluster consists of 200 locations (red cross) with a standard deviation of ~17 nm, similar to the clusters in Fig. 2A. The center of each cluster is shown as a blue dot. In the middle panel, each location is rendered as 2D Gaussian distribution ($\sigma \sim 17$ nm). The right panel shows the cross-sectional profiles along two clusters horizontally. Scale bars: 100 nm. See more details in Methods.

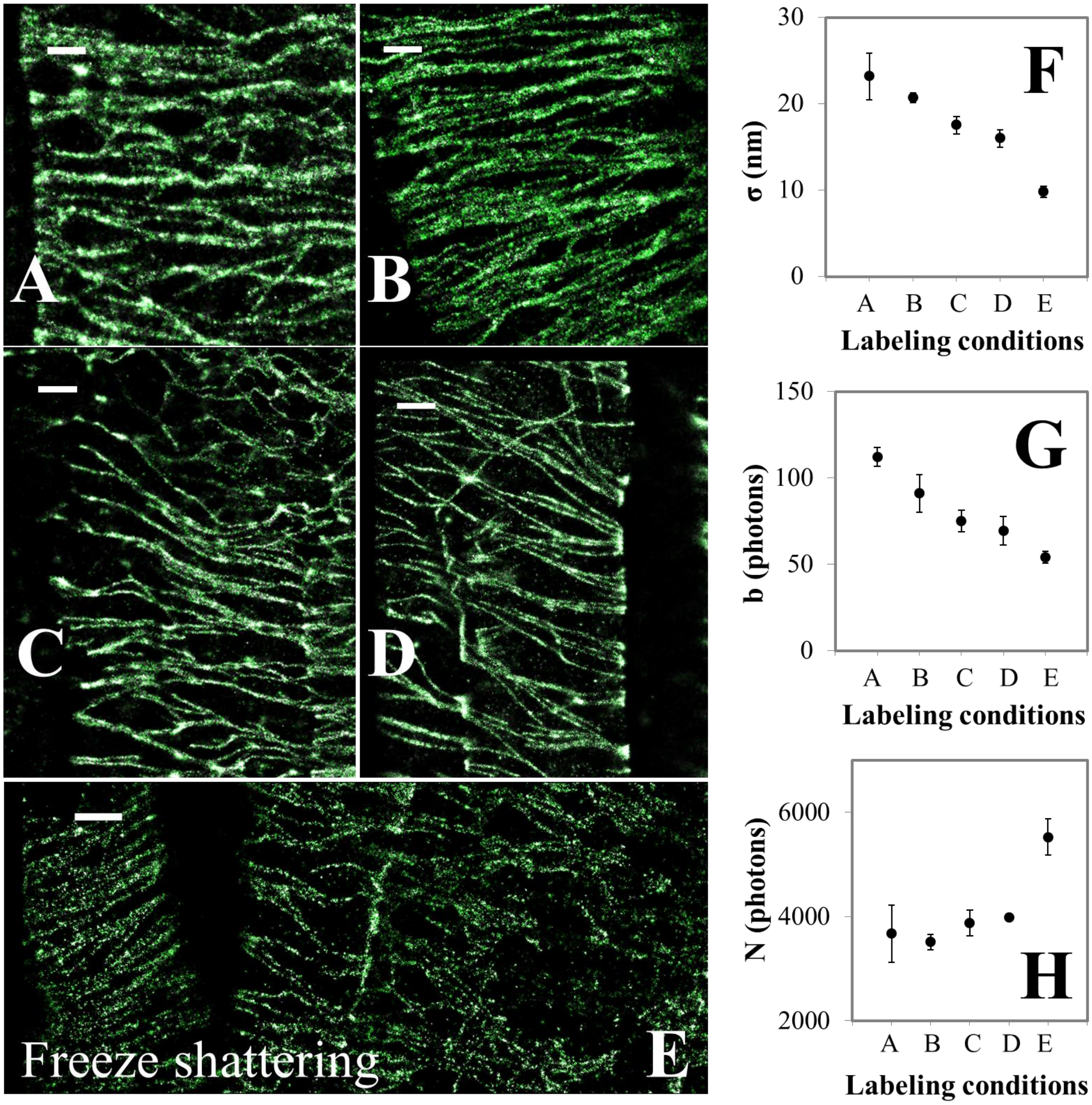

Supplemental Figure 6. Proper labeling density of cortical microtubules by antibody-conjugated dye molecules is critical for generating super resolution images with sub-50nm-spatial resolution. Cortical microtubules in Arabidopsis root cells were first labeled with mouse anti-α-tubulin antibody, followed by goat anti-mouse F(ab')$_2$ fragments conjugated with Alexa Fluor 647. (A)-(D) STORM images using various concentrations of primary antibody and secondary antibody fragments are shown (see table below). (E) STORM image with sample preparation using the freeze-shattering process and with antibody concentrations the same as

those in (D). The corresponding localization precisions using clusters located between cortical microtubules (F), the local background in photon counts around the cluster (G) and the collected photon number of localized positions in the clusters (H) are shown. Scale bars: 1 μm.

	A	B	C	D
primary antibody (μg/mL)	2.0	1.0	2.0	1.0
secondary antibody (μg/mL)	0.20	0.20	0.04	0.04

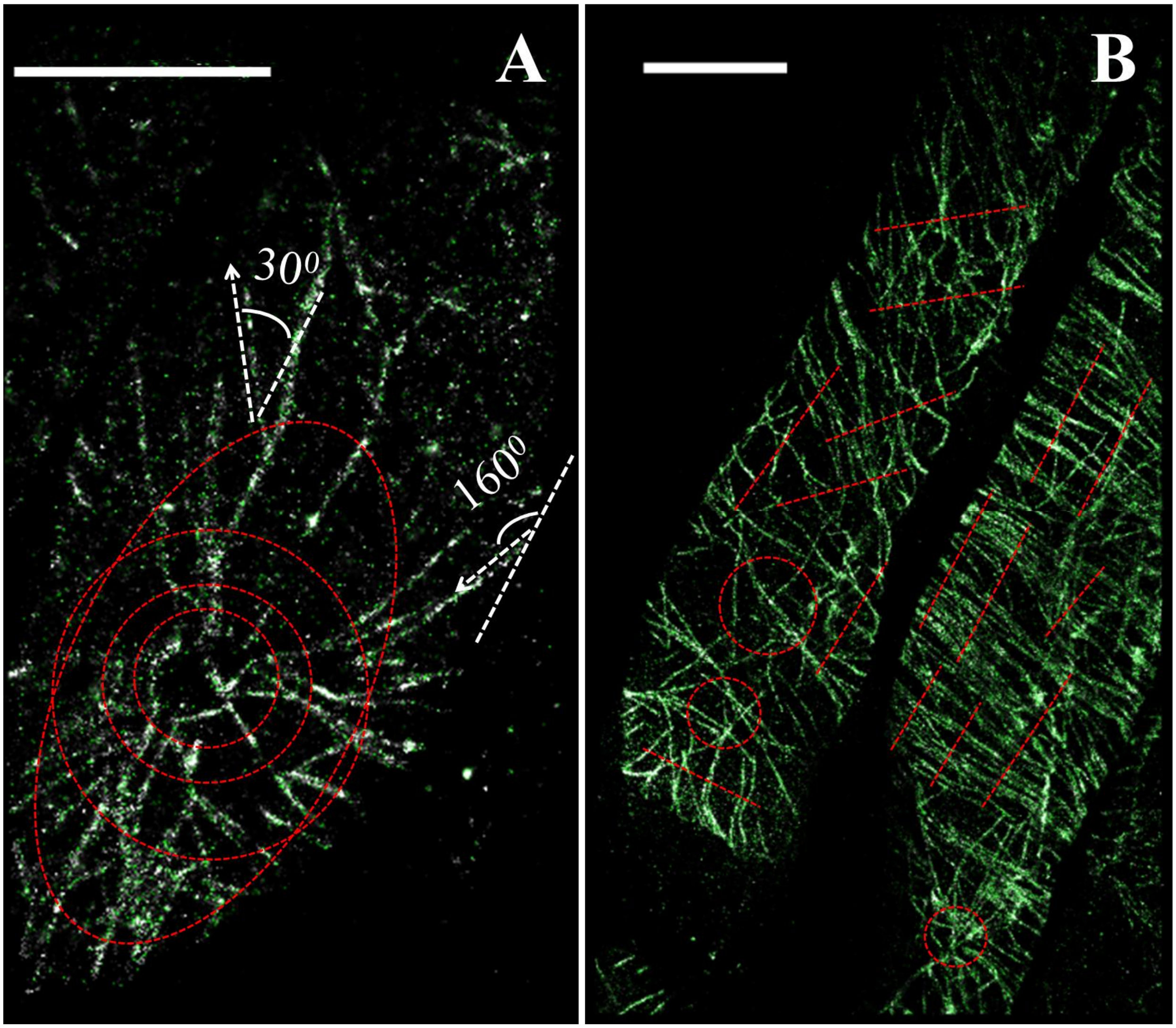

Supplemental Figure 7. Quantitative information on the cortical microtubule network is readily available with a resolution of 20 – 40 nm in STORM images. (A) White dashed lines show the long axis of the plant root cell and white dashed arrows indicate the orientation of individual microtubule filaments. The orientation angles are taken as the angle of each microtubule filament relative to the long axis of the cell. Two examples of microtubule filaments are shown with orientation angle of 30° and 160° respectively. (B) These images demonstrate how the densities of cortical microtubules are measured. Since close microtubule filaments are easily resolvable, the densities can be determined by counting numbers of filaments along the cross-sectional or circular profiles (red dashed lines) depending on the local arrangement of the cortical microtubule network. Scale bars: 5 μm.

Supplemental Table 1. T-test of densities of cortical microtubules between adjacent regions (***a-g***) highlighted in Fig. 5. Cells or regions with densities of cortical microtubules not significantly different (P > 0.1) are highlighted in light blue in the table.

P-value	***a***	***b***	***c***	***d***	***e***	***f***$_1$	***f***$_2$	***g***$_1$	***g***$_2$
a		.0011							
b			.0059						
c				0.1767					
d					0.0001				
e						.6499	.0011		
f$_1$							.0025	.0001	.0001
f$_2$								.2499	.7527
g$_1$									.2220
g$_2$									
